# Peculiarities in thermal transport of nanostructured silicon arrays with different morphology

**DOI:** 10.1038/s41598-025-13379-4

**Published:** 2025-08-26

**Authors:** Lesia Chepela, Pavlo Lishchuk, Isibert Marcel Nkenfack, Viktor Mandrolko, Hadrien Chaynes, Andrey Kuzmich, Poting Liu, Mykola Borovyi, David Lacroix, Vladimir Sivakov, Mykola Isaiev

**Affiliations:** 1 Taras Shevchenko National University of Kyiv, 64/13, Volodymyrska Street, Kyiv, 01601 Ukraine; 2 Université de Lorraine, CNRS, LEMTA, 54000 Nancy, France; 3 Leibniz Institute of Photonic Technology, Member of the Leibniz Research Alliance, Leibniz Health Technologies, Albert-Einstein-Straße 9, 07745 Jena, Germany

**Keywords:** Materials science, Nanoscience and technology, Physics

## Abstract

This study explores the thermal conductivity of nanostructured porous silicon with different morphology produced by metal-assisted chemical etching of silicon wafers with different dopants, doping levels and crystallographic orientation. The wide range of morphological structures observed in the samples strongly depends on the initial wafer characteristics, a factor that cannot be neglected. While previous studies have demonstrated the qualitative capabilities of photoacoustic and Raman spectroscopy in characterizing nanostructured silicon, our work highlights the quantitative discrepancies that can arise when combining these techniques to investigate thermal properties. The differences in the results obtained using these methods can be attributed to the distinct nature of the information they provide: photoacoustic spectroscopy probes the effective thermal conductivity over larger areas, whereas Raman spectroscopy offers localized measurements. Furthermore, our Monte Carlo simulations provide insights into the morphological features of porous silicon that influence the interpretation of experimental data. This study underscores the importance of a comprehensive approach, combining both experimental and theoretical methods, to accurately assess the thermal transport properties of nanostructured materials.

**Supplementary Information:**

The online version contains supplementary material available at 10.1038/s41598-025-13379-4.

## Introduction

 Functional silicon-based nanostructures play a crucial role in modern advancing green energy solutions. Among these, porous silicon-based materials stand out for their potential in photovoltaics^1,2^ thermoelectric devices^3^, and hydrogen storage systems^4,5^. Efficient thermal management is essential to mitigate overheating and hotspots in these applications, underscoring the importance of understanding the thermal transport properties of these materials.

Nanostructured porous silicon (nSi) exemplifies a promising material for green energy technologies^6^. Their high surface-to-volume ratio is particularly beneficial for enhancing the performance of solar cells by facilitating solar light absorption and energy conversion. However, arrays of nSi exhibit reduced thermal conductivity compared to bulk silicon due to increased phonon scattering at the Si` surface. While this reduced thermal conductivity can pose challenges related to local overheating, it can also be advantageous for alternative energy solutions^7^. It is well-known that nSi arrays can adjust thermal properties by modifying wire diameter, its roughness, the distance between them, and material composition^8–11^. This offers the flexibility for tuning its thermal performance to the specified applications by varying the conditions of the fabrication and parameters of the initial substrate. Therefore, gaining insights into the thermal properties of nSi is crucial for optimizing their efficient use in various energy applications. Numerous approaches were developed to study the thermal transport properties of nanostructured materials. Among them, we can mention scanning thermal microscopy (the use of a sharp tip allows us to measure local temperature fluctuations on the surface of the material)^12,13^, 3-Omega method (application of a high-frequency heating source to the nanostructure and analysis of the resulting temperature response)^14,15^, hot disk method (using a thin disk-shaped sensor)^16^, thermoreflectance method (a technique that uses the change in the reflectance of a material’s surface as a function of temperature)^17,18^, thermal bridge method (a method that measures the temperature difference between two points on a sample)^19^, etc. Additionally, as supplementary structural characterization methods, one can mention X-ray and neutron scattering, which are useful for gaining insight concerning the phonon dispersion relation and the density of states^20,21^. These methods play crucial roles in the field of nanotechnology and materials science, helping to understand and manipulate the thermal transport properties of nanostructured materials. They contribute to developing advanced materials for various applications, including electronics, thermoelectrics, and heat management in devices.

Photothermal methods share the common attributes of being contactless and non-destructive, making them valuable tools for studying nanomaterials. For instance, photoacoustics involves irradiating the sample with modulated or pulsed light, leading to a temperature rise as the light energy is converted into heat. This temperature increase causes the sample to expand, generating acoustic waves that can be detected by acoustic sensors and converted into an acoustic signal^22–28^. In contrast, Raman spectroscopy relies on the inelastic scattering of monochromatic light directed at the sample^29–31^. The features of the scattering light, such as the position of the Raman modes and/or Stokes-Antistokes ratio can be used to determine the magnitude of the local heating caused by laser radiation.

In this study, we used both Raman scattering and photoacoustic techniques to investigate the thermal properties of nSi fabricated under various conditions. Our findings demonstrate that combining these methods can give us deeper insights into the complex thermal transport mechanisms in nanostructured systems. Moreover, we emphasize that the measured thermal conductivity values in nanostructured silicon-based systems may be influenced by the specific configuration of the experimental setups. Thus, a combined approach involving experimental and theoretical methods is essential to comprehensively understand thermal transport in nanostructured silicon.

## Materials and methods

### Fabrication of nSi array

**Fig. 1:**
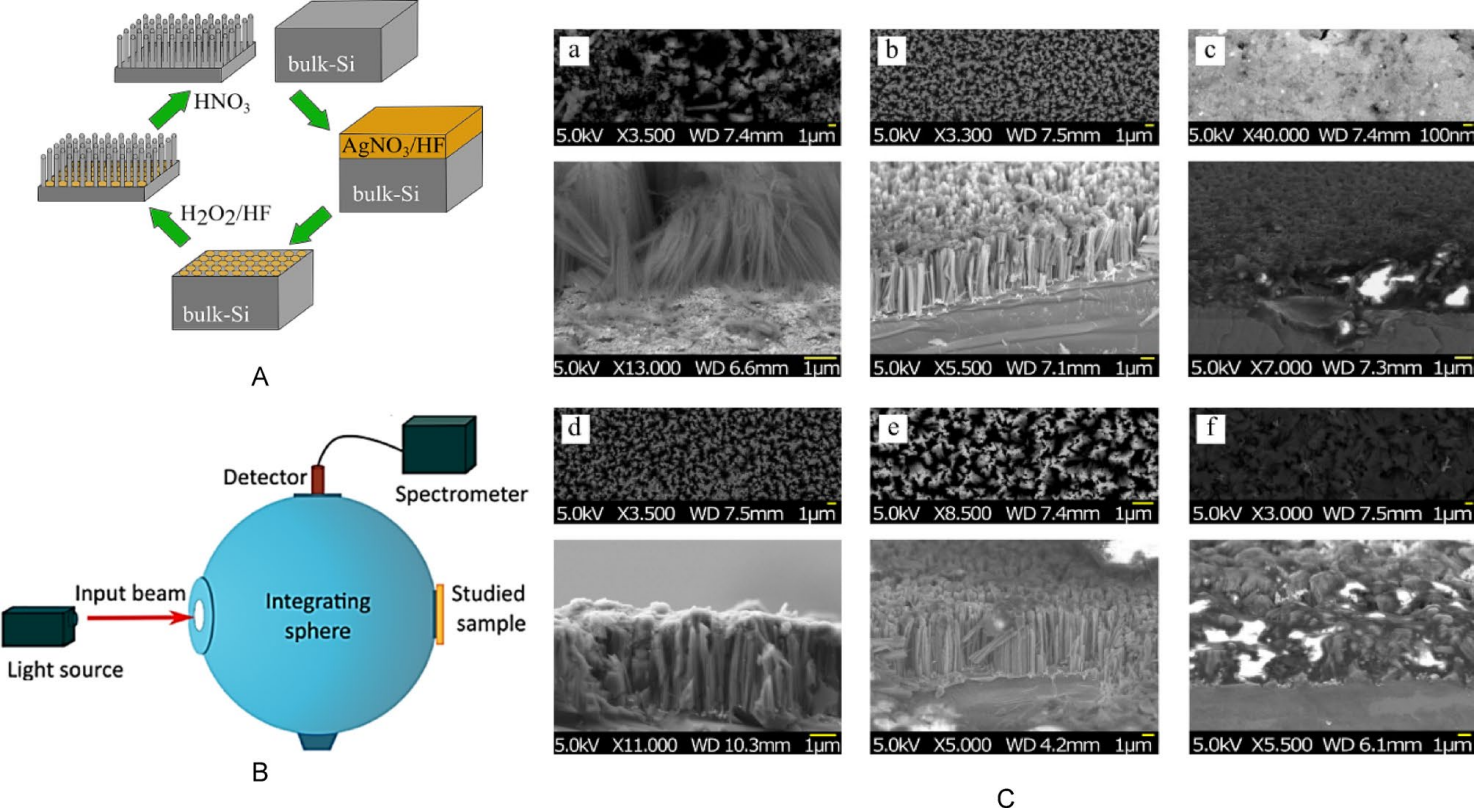
Schematic representation of the fabrication of nSi by the MACE method (A); schematic sketch-view of the setup with the integrating sphere in the configuration of the regime of total reflectance measurement (B); SEM images of the studied samples a-f (see Table 1). Top image: a planar view of the sample’s surface, Bottom image: a cross-sectional view of the samples (С).

Nanostructured Si samples were fabricated by metal-assisted chemical etching (MACE) of (100), (101), and (111)-oriented p-type and n-type c-Si wafers with resistivity from 0.001 Ω cm to 20 Ω cm. The MACE process is schematically presented in Fig. 1A. The wafers were washed with a 2% HF solution for 1 min to remove the native oxide before the MACE procedure^32^. The first stage of MACE involved the deposition of polycrystalline silver nanoparticles with different morphologies on the surface by immersing them in an aqueous solution of 0.01 M silver nitrate (AgNO_3_) and 5 M HF in a volume ratio of 1:1 for 15–30 s. In the next step, the c-Si wafers covered with Ag nanoparticles were immersed in a solution containing 5 M HF and 30% H_2_O_2_ in a volume ratio of 10:1 for 20 min (Table 1). Finally, the samples were washed with deionized water and dried at room temperature. The morphology of achieved nanostructured surfaces was performed using scanning electron microscopy. The thickness of the etched samples was determined from 2 to 5 μm, as shown in Fig. 1C.

We used the MACE process, which typically results in samples with an ordered array of nanowires, as seen in the a, b, d, and e series samples. The orderly arrangement of nanowires may have been somewhat disrupted during the mechanical preparation of the samples for determining the height of the nanostructured layer in the SEM images. However, there are also samples with sponge-like thin films, clearly lacking anisotropy or order in morphology, observed in the c and f series.

### Optical properties

Measurements of diffuse reflectance can be obtained, as depicted in Fig. 1B. In the latter approach, using an integrating sphere is a standard method to assess the optical properties of various samples^33,34^ including silicon nanowires^35^. This method serves as a valuable tool for quantifying the total diffuse reflectance of the sample`s surface. The integrating sphere is a spherical hollow chamber coated with a highly diffusive reflective material, guaranteeing the reflection of all incoming light within it.

In such experiments, the radiation an IR detector detects is directly related to the scattering light. In our experimental setup, we employed a UPG-150-ART integrating sphere of 150 mm diameter gold-coated. In this study, we measured the total diffuse reflectance for each sample at a wavelength of 532 nm. The achieved coefficients of total reflection are presented in Table 1. The uncertainty of measurements was determined by conducting repeated measurements and analyzing the standard deviation of the results.

**Table 1:** Structural and optical parameters of the fabricated nSi samples.

№	Wafer	Orientation	Doping	*R*_es_ (Ω·cm)	Doping Molar fraction	Thickness, µm	Reflection coefficient
a	n-Si	111	As	0.001–0.002	0.001–0.002	~ 3	0.1345 ± 0.0003
b	n-Si	101	Sb	0.015–0.020	3·10^−5^ – 0.0014	~ 4.5	0.1073 ± 0.0003
c	p-Si	100	B	< 0.005	< 5·10^−4^	~ 2.6	0.1040 ± 0.0003
d	p-Si	100	B	10–20	1.6·10^−8^ – 3·10^−8^	~ 4	0.1602 ± 0.0003
e	n-Si	100	P	0.005–0.02	3·10^−5^ – 3·10^−4^	~ 5	0.1288 ± 0.0003
f	n-Si	111	P	< 0.005	< 3·10^−4^	~ 5	0.1062 ± 0.0003

### Photoacoustic measurements

**Fig. 2:**
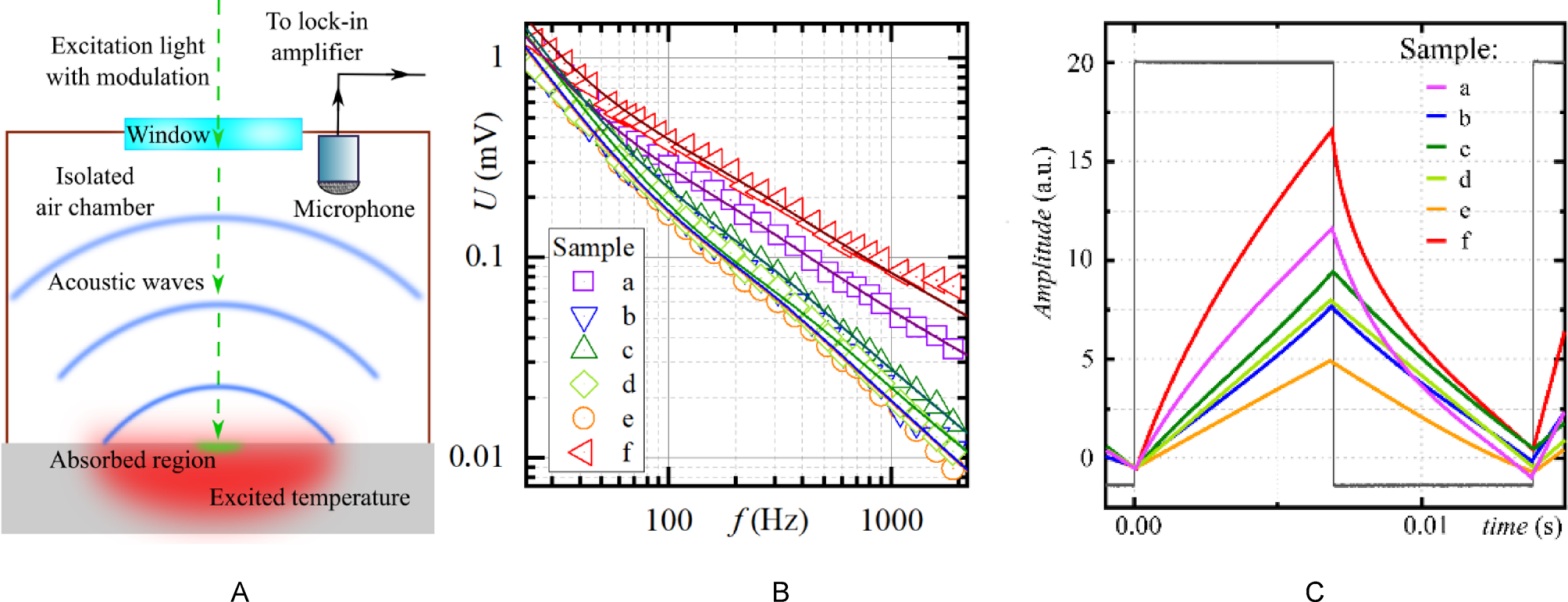
Schematic representation of the photoacoustic setup used in current work (A), amplitude-frequency dependences of PA signal for samples 1–6 (solid lines present the results of the simulation) (B), the photoacoustic signal waveforms of the samples scaled to the same amplitude and a square modulated reference signal for visualization purposes (C).

The thermal transport properties were evaluated using the photoacoustic (PA) gas-microphone technique in a conventional configuration (Fig. 2A)^36^. As the excitation source a 100 mW green laser (λ = 532 nm) was used. An optical system homogeneously distributed light radiation on the sample inside the PA cell. This created a focused spot with a 3 mm diameter on the sample`s surface. The modulation of the current of the power source performed the modulation of the light intensity. At the given wavelengths, light was absorbed within the upper layer of the samples. A Panasonic WM-61 microphone positioned adjacent to the gas chamber measured the generated PA signal.

The measured PA signal was analyzed using a lock-in amplifier, where the signal from the power source was a referenced one. This vital step significantly enhanced the signal-to-noise ratio, allowing for precise measurements of the amplitude-frequency characteristics (AFC) within the 20–2000 Hz range (Fig. 2B). Both the photoacoustic and reference signals were also displayed on an oscilloscope (Fig. 2C).

We performed numerical simulations based on a well-established theoretical framework to better understand the experimental PA response and extract quantitative information about the material’s thermal properties. The simulations employed a model initially developed by Rosencwaig and Gersho, extensively used for PA signal analysis in various material systems^37^. Central to this model is the calculation of the variable temperature component (*θ*) within the sample:

1 $$\:\frac{d}{dz}\left(k\left(z\right)\frac{d\theta\:}{dz}\right)-2\pi\:fc\rho\:=I\left(1-R\right)\alpha\:{e}^{-\alpha\:z},$$

where *k* is the cross-plane thermal conductivity, *c* is the heat capacity, *ρ* is the mass density, *f* is the laser modulation frequency, *I* is the amount of absorbed light by the structure, *α* is the optical absorption coefficient, and *R* is the reflectivity of the sample surface.

In our study, we considered a two-layered structure, with each layer possessing distinct properties, such as thermal conductivity, heat capacity, density, and optical absorption.

We made the following assumption to simplify the theoretical consideration of the process of signal formation: (1) the absence of heat outflows from the boundaries of the two-layer structure, ensuring a confined thermal system, (2) continuity of temperature and heat fluxes across the interface between the layers, ensuring a smooth transfer of thermal energy.

Once the temperature field is calculated, the model enables the evaluation of pressure fluctuations within the PA cell. These fluctuations, which constitute the simulated PA signal, are calculated using a formalism derived from the Rosencwaig-Gersho framework^26,38^:

2 $$\:\rho\:\left(\omega\:\right)\:\sim\underset{0}{\overset{-\infty\:}{\int\:}}\theta\:\left(0\right)\cdot\:{exp}\left(\sqrt{i\raisebox{1ex}{$\omega\:{c}_{air}{\rho\:}_{air}$}\!\left/\:\!\raisebox{-1ex}{${k}_{air}$}\right.}z\right)dz=-\theta\:\left(0\right)\sqrt{\frac{{k}_{air}}{i\omega\:{c}_{air}{\rho\:}_{air}}},$$

where index “air” means that the following parameters belong to the gas.

In this method, the thermal conductivity value was determined by adjusting the experimental and theoretical curves to achieve the best correlation between them, with the effective thermal conductivity of the layer being fine-tuned accordingly. The measurement uncertainty for this method was based on the values at which qualitative matching of the curves was achieved, encompassing instrumental factors and other sources of error.

### Raman measurements

**Fig. 3:**
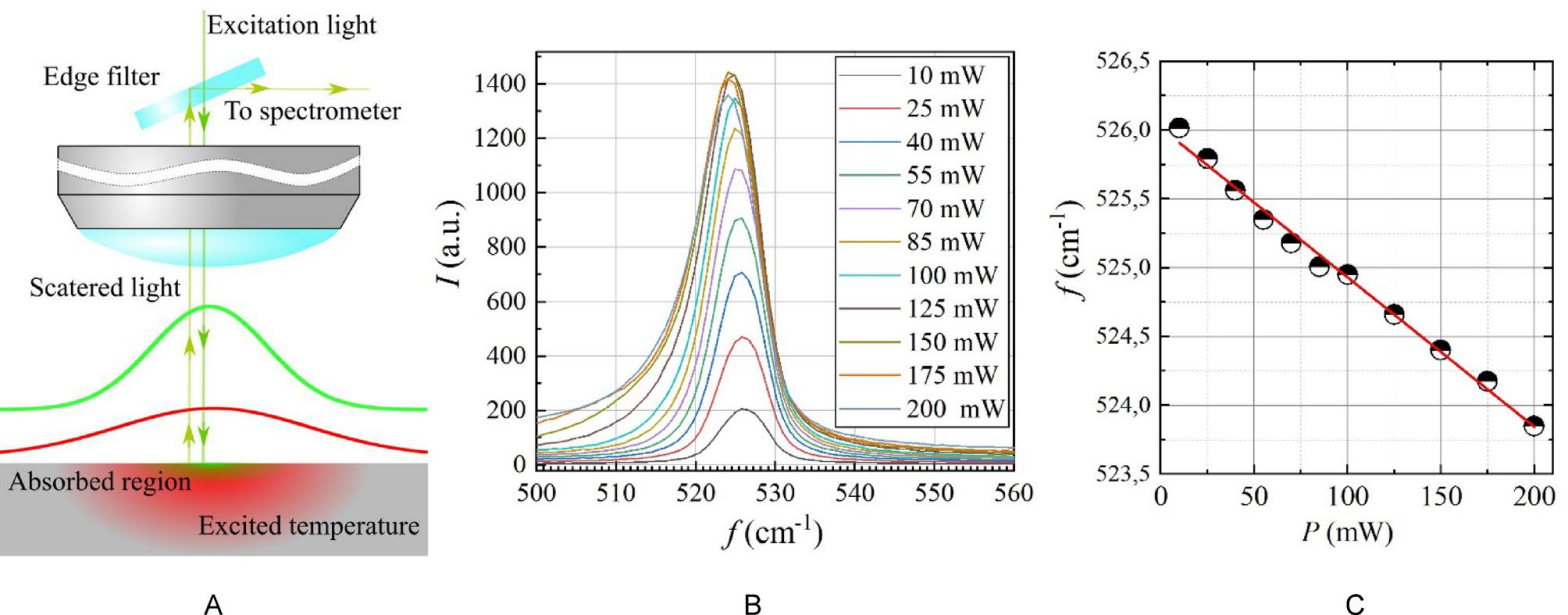
Schematic sketch view of the experimental configuration (A). Raman spectra (B) and dependence of the Raman peak position on laser power (C) of 5 μm thick nSi sample (*α*=−0.01156 ± 0.0003 cm^−1^/mW).

For the characterization of the thermal transport properties of the nSi samples, the Raman approach was used. The laser with a wavelength equal to 532 nm was used as the excitation source. The radiation from the light source was focused on the sample surface with the optical system to form the laser spot with a circular shape and radius equal to 10.5 μm. The scattered light was collected by the optical system and analyzed by the spectrometer Princeton Instruments as shown in Fig. 3 (A). The dependence of the peak position on the laser power was measured within the range of 10 to 200 mW (Fig. 3 (B)). The acquired spectra were fitted with Gaussian curves to determine the spectral positions of the peaks and their relationship with laser power (Fig. 3 (C))^39^.

For the evaluation of temperature rise, we used the linear coefficient of the dependence of the Raman peak position on the temperature from literature data *β* = − 0.023 ± 0.002 cm^−1^/K^40^: These slopes were utilized to calculate the thermal resistance of each sample.

3 $$R=\theta /p,$$

where is the temperature rise, and p is the laser power.

To evaluate the thermal conductivity, the following heat conduction equation was considered:

4 $$\:\overrightarrow{\nabla\:}\left(\widehat{k}\overrightarrow{\nabla\:}\theta\:\right)+s\left(\overrightarrow{r}\right)=0,$$

where $\:\widehat{k}$ is the thermal conductivity tensor^41^, $\:\:s\left(\overrightarrow{r}\right)$ is the laser-induced volumetric heating source^41^.

For nSi, the thermal conductivity tensor includes *ki*_*n−p*_ and *k*_*cr−p*_, which are the in-plane and cross-plane thermal conductivities, respectively. In this case, the boundary conditions mentioned above for PA measurements were supplemented by the condition of the absence of temperature perturbation at a large distance from the laser source.

**Fig. 4:**
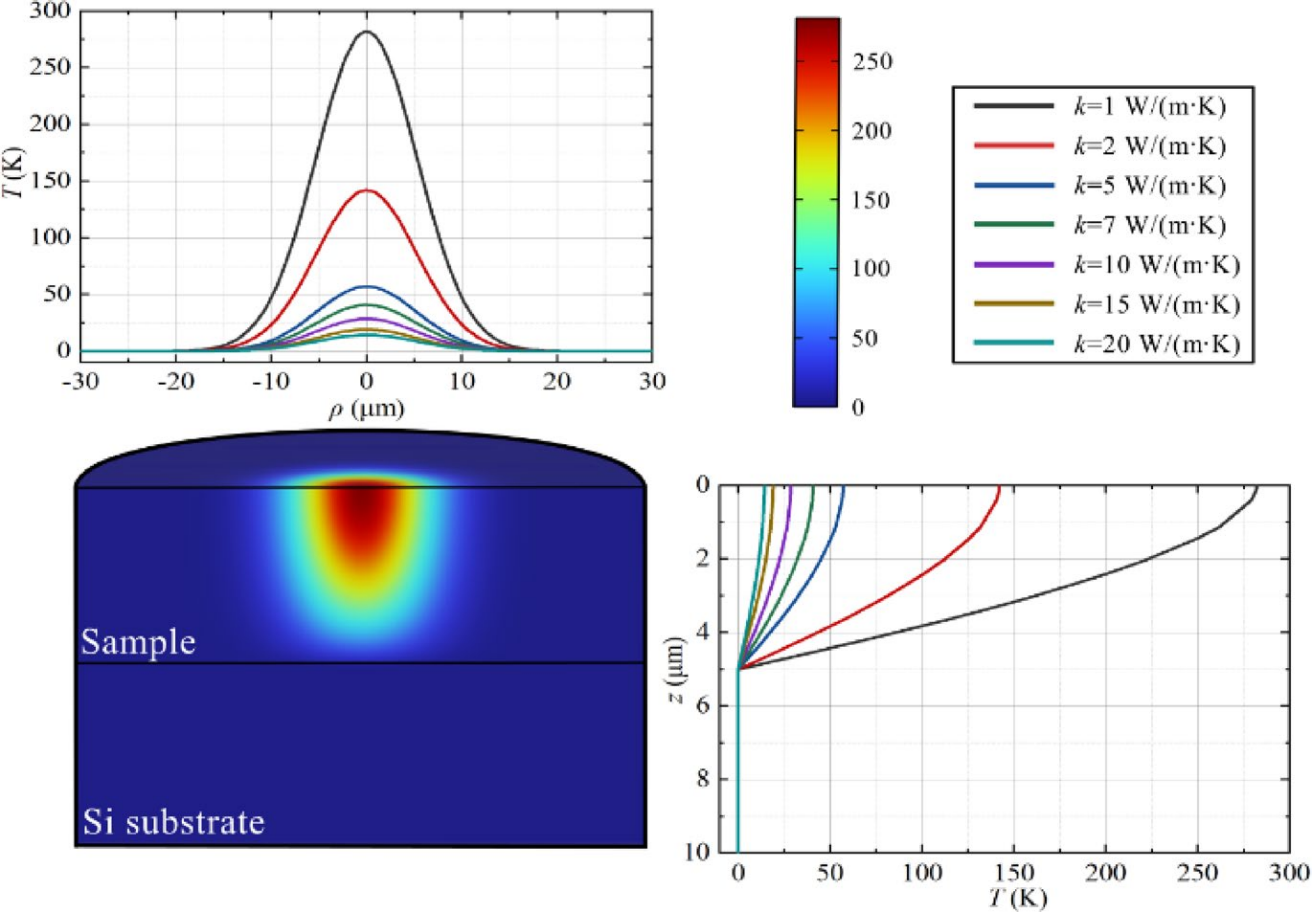
Temperature profiles for sample “e” at different values of the cross-plane thermal conductivity: on the surface of the nSi sample (A); depth of the nSi sample (B).

An effective medium model was used to simulate porous layer thermal conductivity. The anisotropic approximation was evaluated using Comsol Multiphysics^42^. The average temperature of the anisotropic (*ki*_*n−p*_ ≠ *k*_*cr−p*_) and isotropic (*ki*_*n−p*_ = *k*_*cr−p*_) approximations was calculated as a function of the thermal conductivity for each studied sample (Fig. 4).

The optical absorption coefficient for the nSi layer was calculated using the equation *α = α*_*Si*_ *(1 – P)*, where *P* is the porosity of the sample, and *α*_*Si*_ = 7.85 × 10^3^ cm^−1^ is the optical absorption coefficient of crystalline silicon.

**Fig. 5:**
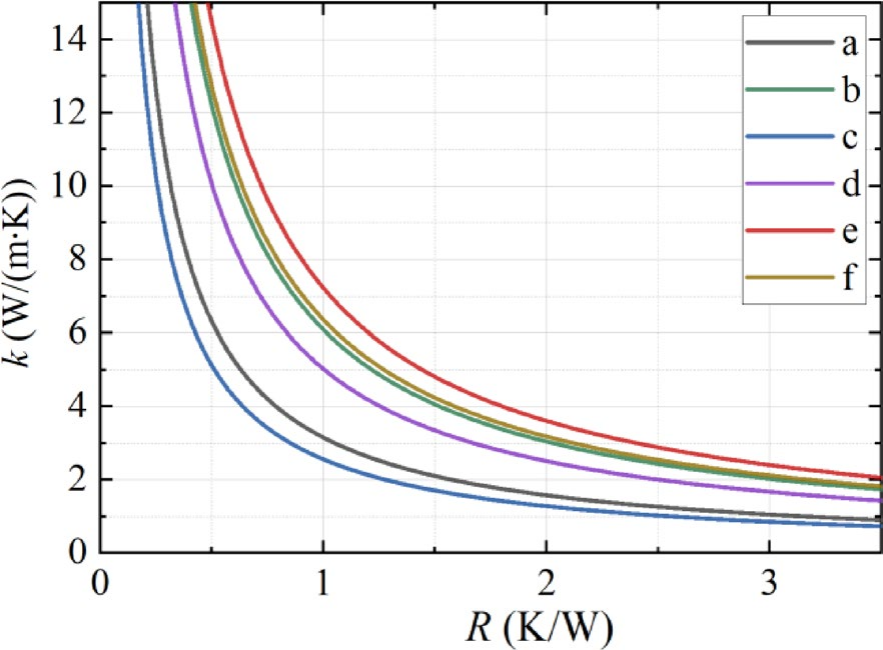
Dependencies of thermal resistance on the value of the coefficient of thermal conductivity for all samples.

Ultimately, the thermal conductivities of the analysed samples can be determined by establishing a connection between the calculated and experimentally measured thermal resistance (〈*θ*〉/p) (see Fig. 5), where 〈*θ*〉 – is an averaged temperature. The thermal conductivity values are derived by fitting the experimental data to the results obtained through simulations described in detail in reference^41^.

In the study, the accuracy of peak determination was the basis for subsequent calculations of thermal conductivity. The error was determined by calculating the mean thermal conductivity from multiple measurement points, and computing the standard deviation and the standard error to assess the reliability of the mean value. Furthermore, the z-score was applied to evaluate the deviations of individual measurements from the mean, determining the statistical significance of these deviations.

### Monte Carlo methods

Adopting a particle-based approach, the dynamics of phonon transport is described by the Boltzmann Transport Equation (BTE). In this work, we solve this equation using the Monte Carlo method combined with the Green-Kubo formalism (MC-GK), while applying the relaxation time approximation^43^. Phonons are considered heat-carrying particles with well-defined frequencies and polarizations. They are treated as classical energy packets, moving and colliding within the nanostructure over time. The method used is detailed in references^44,45^.

5 $$\:\frac{df}{dt}+{\nabla\:}_{K\omega\:}.{\nabla\:}_{r}f=\frac{({f}^{0}-f)}{\tau\:(\omega\:,T)},$$

where $\:f(K,P,\:r,t)$ describes the probability of finding a heat carrier with a specific energy and wave vector K at a given position r in space. The first term (on the left) of the Boltzmann Transport Equation (BTE) corresponds to the “transport” term of the phonon, while the second term (on the right) characterizes the intrinsic scattering mechanisms with the relaxation time, defined by:

6 $$\:{\tau\:}^{-1}={\tau\:}_{I}^{-1}+{\tau\:}_{N}^{-1}+{\tau\:}_{U}^{-1},$$

$\:{\tau\:}_{I}$, $\:{\tau\:}_{N}$, $\:{\tau\:}_{U}$ are respectively the relaxation time for diffusion by isotope and impurities, or by ``Normal’’ and ``Umklapp’’ processes, they incorporate interactions involving three-phonon processes. Expressions of relaxation times are given by:

7 $$\:\begin{array}{c}{\tau\:}_{I}^{-1}={(A}_{iso}+{A}_{doped}){\omega\:}^{4}\\\:{\tau\:}_{LN}^{-1}={B}_{LN}{\omega\:}^{2}{T}^{3}\:;\:{\tau\:}_{TN}^{-1}={B}_{TN}\omega\:{T}^{4}\\\:{\tau\:}_{LN}^{-1}={B}_{LU}{\omega\:}^{2}T{e}^{\frac{{\theta\:}_{L}}{3T}}\:;\:{\tau\:}_{TN}^{-1}={B}_{TU}{\omega\:}^{2}T{e}^{\frac{{\theta\:}_{T}}{3T}}\end{array},$$

Each scattering process has its expression, depending on frequency and temperature. The constants $\:{(A}_{iso},\:{B}_{LN},\:{B}_{TN},\:{B}_{TU},\:{B}_{LU})\:$ are obtained by fitting experimental curves of the thermal conductivity of the bulk^46^ material as a function of temperature. Table 2 lists these constants for the ``Normal”, ``Umklapp’’ and ``Impurity’’ scattering processes.

**Table 2:** Relaxation time parameters for Si for the Debye–Callaway model.

Relaxation time parameters	Values (Units)
$\:{A}_{iso}$	$\:1.32\:\times\:{10}^{-45}\:\left({s}^{3}\right)$
$\:{B}_{LN}$	$\:0.8\times\:{10}^{-24}\:\left({sK}^{-3}\right)$
$\:{B}_{TN}$	$\:2.0\times\:{10}^{-13}\:\left({K}^{-4}\right)$
$\:{B}_{LU}$	$\:3.0\times\:{10}^{-20}\:\left(s{K}^{-1}\right)$
$\:{B}_{TU}$	$\:8.3\times\:{10}^{-20}\:\left(s{K}^{-1}\right)$
$\:{\theta\:}_{L}$	583.8 (K)
$\:{\theta\:}_{T}$	230.0 (K)

These constants cannot be deduced directly from analytical expressions, as phonon group velocities, which are not constant and are deduced from phonon dispersion properties (quadratic fitting) suggested by Pop and Dutton^47^, the dispersion properties of the silicon used in this work are derived from parabolic fits defined by: $\:\omega\:={c}_{P}{K}^{2}+{v}_{p}K;\:{c}_{P},\:{v}_{p}$ parameters are detailed in the Table 3.

In this work, we used the following model^48,49^ to calculate the relaxation time parameter associated with silicon doping:

8 $$\:{A}_{doped}=\frac{V\:{ \Gamma }_{doped}}{4\pi\:{v}_{s}^{3}},$$

$\:{ \Gamma}_{doped}=y\left({\left(\frac{\delta\:M}{M}\right)}^{2}+{{\:\epsilon\:}^{2}\left(\frac{\delta\:R}{R}\right)}^{2}\right)\:$ is the doping parameter where, y is the molar dopant fraction, $\:\:\frac{\delta\:M}{M}\:$ is the ratio between the atomic mass variation between silicon atoms and dopant atoms $\:\left(\delta\:M\right)\:$ to the atomic mass of silicon $\:\left(M\right),\:\frac{\delta\:R}{R}\:$ is the ratio of the change in the atomic radius between silicon and dopant ($\:\left(\delta\:R\right)\:$ to the atomic radius of silicon $\:\left(R\right)$, the parameter ε depends on how the nearest and farthest bonds combine in the scattering^48^; in our case, it is equal to^42^ in all our calculations, which allows us to obtain a satisfactory estimate of the thermal conductivity, $\:{v}_{s}=\:\frac{3}{\frac{1}{{v}_{LA}}+\frac{2}{{v}_{TA}}}$ is the speed of sound in the material and $\:V$ is the volume of the silicon crystal lattice. In the case of sample № d, to improve the estimation of thermal conductivity via the numerical method (MC-GK), it was necessary to use an additional parameter of relaxation time specific for diffusion $\:{\varvec{A}}_{\varvec{x}}$. This sample, presenting a high electrical resistance, could have undergone volume defects during its manufacture. The parameter $\:{\varvec{A}}_{\varvec{x}}\:$ can thus be interpreted as reflecting the impact of these volume defects.

**Table 3:** Data used to fit Si dispersion curves.

	(Units)	Orientation (100)	Orientation (111)
$\:{c}_{LA}$	$\:\times\:{10}^{-7}\left({m}^{2}{s}^{-1}\right)$	−2.22	−8.88
$\:{c}_{TA}$	$\:\times\:{10}^{-7}\left({m}^{2}{s}^{-1}\right)$	−2.28	−7.10
$\:{v}_{LA}$	$\:\times\:{10}^{-3}\left(m{s}^{-1}\right)$	9.26	17.50
$\:{v}_{TA}$	$\:\times\:{10}^{-3}\left(m{s}^{-1}\right)$	5.24	8.20

**Table 4:** Characteristics of the obtained nanostructures (nanowires (NW) arrays or porous films) as well as the relaxation time parameters of each sample, determined using the model and used in the simulations.

№	Nanostructures used in simulations	Doping	Molar fraction (y)	$\:{\varvec{A}}_{\varvec{d}\varvec{o}\varvec{p}\varvec{e}\varvec{d}}$ $\:\left({\varvec{s}}^{3}\right)$	$\:{\varvec{A}}_{\varvec{x}}$ $\:\left({\varvec{s}}^{3}\right)$
a	NW with diameter ~ 65 nm, length ~ 3 μm	As	0.002	9.57·10^−44^	0
b	NW with diameter ~ 105 nm, length ~ 4.5 μm	Sb	0.0014	4.60·10^−42^	0
c	Film with thickness ~ 2.6 μm	B	5·10^−4^	1.59·10^−42^	0
d	NW with diameter ~ 65 nm, length ~ 4 μm	B	3·10^−8^	9.58·10^−47^	8·10^−44^
e	NW with diameter ~ 190 nm, length ~ 5 μm	P	8·10^−5^	7.46·10^−44^	0
f	Film with thickness ~ 5 μm	P	3·10^−4^	6.50·10^−44^	0

Table 4. The value of the relaxation time parameter of impurity scattering and nanostructure characteristics.

Solving the Boltzmann transport equation (BTE) by the (MC-GK) method is an iterative process that includes (i) an initialization phase, (ii) the movement of heat carriers through the structure, taking into account potential boundary reflections. In our case, we model diffuse reflections. For this, the reflections are simulated by selecting a random vector $\:{\varvec{R}}_{\varvec{r}}$ in the half-hemisphere oriented along the normal to the collision surface given by the relation:

9 $$\:{\varvec{R}}_{\varvec{r}}=\text{cos}\left(\theta\:\right).\varvec{n}+\text{sin}\left(\theta\:\right).\left(\text{cos}\left(\phi\:\right){\varvec{T}}_{1}+\text{sin}\left(\phi\:\right){\varvec{T}}_{2}\right)\:\:\:\:\:\:\:\:\:\:\:\:\:\:\:\:\:\:\:\:\:\:\:\:\:\:\:\:\:\:\:\:\:\:\:\:\:\:\:\:\:$$

where *θ* lies between [0, π/2]. The value of cos(*θ*) is determined using Lambert’s, where cos(θ) = = $\:\sqrt{{r}_{1}}$, with $\:{r}_{1}$ being a random number between 0 and 1. Similarly, *φ* is calculated as $\:2\pi\:{r}_{2}$, where $\:{r}_{2}\:$ is another random number between 0 and 1. The vectors $\:{\varvec{T}}_{1}$ and $\:{\varvec{T}}_{2}$ are normalized and tangential to the reflection surface, satisfying the conditions $\:{\varvec{T}}_{1}$ ⊥ $\:{\varvec{T}}_{2}$ ⊥ **n**. The process then consists of iii) evaluating the intrinsic carrier scattering probability and iv) calculating properties such as energy, temperature and heat flux. Steps ii) to iv) are repeated for a predefined number $\:{N}_{t}\:$ of time steps. It is important to note that no temperature gradient is imposed in the system. The simulations are performed at a uniform temperature, and the thermal conductivity of the system is evaluated using the “Green-Kubo” (GK) formulation^44^, which determines the thermal conductivity tensor by calculating the flux autocorrelation. This technique has proven to be reliable when it is used to solve the Boltzmann transport equation. More generally MC solution of the BTE when applied to nanowire structures has proven to be flexible for different types of geometries, materials and temperature levels as discussed in former studies^45,50–52^.

**Table 5:** The value of the thermal conductivity of the samples.

№	PA technique: k, W/$\:{\text{m}}^{-1}{\text{K}}^{-1}$	Raman technique: k, W/$\:{\text{m}}^{-1}{\text{K}}^{-1}$	Porosity used MC-GK (%)	Simulation MC-GK: k, W/$\:{\text{m}}^{-1}{\text{K}}^{-1}$
a	0.6 ± 0.1	1.2 ± 0.1	0	2.9 ± 0.1
b	5 ± 1.5	4 ± 1	0	4.1 ± 0.1
c	0.8 ± 0.2	2.7 ± 0.6	40 (0)	3.3 ± 0.1 (4.1 ± 0.1)
d	5 ± 1.5	8 ± 1	0	7.9 ± 0.2
e	7 ± 2	10.5 ± 1.7	0	11.7 ± 0.2
f	0.9 ± 0.1	2.6 ± 0.9	40 (0)	3.9 ± 0.3 (10.8 ± 1)

## Results and discussion

In the PA technique, the thermal conductivity of the samples is tracked by matching the frequency of the change in the slope of the PA signal amplitude in a double logarithmic scale. The thermal conductivity of the top layer of the material determines the slope of the line at high frequencies. The optical absorption coefficient is tracked by matching the amplitude of the simulation to the corresponding parameter on the experimental curve. In this case, the samples’ optical reflectance was considered, previously obtained using the integrating sphere method. This ensures that the measured PA signal is only due to the absorption of the light by the material and not due to reflection from the surface. The optical absorption coefficient for nSi, as in the case of the Raman method, was calculated using the equation *α = α*_*Si*_ *(1 – P)*, where *P* is the porosity of the sample. The corresponding values are given in the Table 5.

The thermal conductivity of the samples was determined by the Raman method both directly from the experimentally obtained thermal resistances and from those determined by model calculations (see above). The experimentally obtained thermal conductivity was used to adjust the initial parameters of the model calculations. The obtained values of the thermal conductivity of the samples are also given in Table 5.

**Fig. 6:**
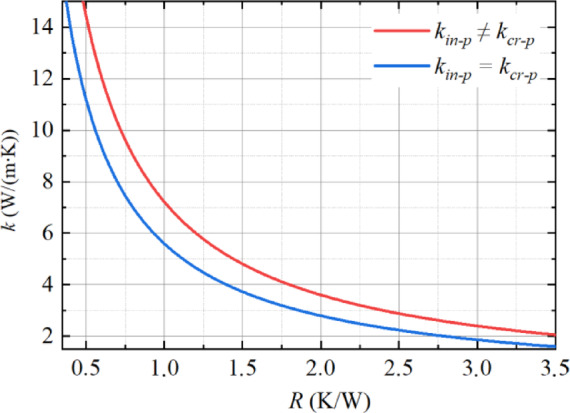
Dependence of thermal conductivity on thermal resistance for sample “e”. The blue line represents the calculation in the isotropic approximation, while the red line represents the anisotropic approximation.

Figure 6 shows the calculated dependence of thermal conductivity on thermal resistance for sample “e” in two approximations. The comparison demonstrates that the thermal conductivity values in the isotropic approximation are 25–30% lower than in the anisotropic approximation.

As can be seen from the table, samples b, d, and e have the highest thermal conductivity. These samples have a well-ordered structure of the silicon nanowires perfect structure of the quantum wire arrays (see SEM images). These arrays are characterized by significant spatial structure anisotropy between the in-plane and cross-plane directions, hence, the anisotropy of the thermal conductivity *k*_*cr-p*_ *> k*_*in-p*_, which was taken into account in the calculations by the Raman spectroscopy method. For samples c and f, the thermal conductivity values are 2–4 times lower than for samples b, d, and e. SEM images of these samples demonstrate a noticeably fractal-like structure quality of their structure. This leads to less pronounced anisotropy of thermal transport properties. In particular, sample f was assumed to be isotropic in the calculations (*k*_*cr-p*_ *= k*_*in-p*_), which allowed us to approximate the value of its calculated thermal conductivity to the experimentally determined one. Thus, the sample (a) has the lowest thermal conductivity. SEM images of this sample show that its morphology differs from that of other samples - the silicon nanowires are thinner. Furthermore, the distance between separate nanowires is higher, and they are much denser on the substrate than in other samples. In addition, one can observe the areas of fusion of the separate nanowires on the top of the Si NWs array. This may arise due to the lowest specific electrical resistance of the initial wafer.

It should be noted the qualitative correlation between the results analyzed by model calculations using the Raman method and the photoacoustic method. However, some divergence between experimental results obtained by these two approaches is presented. These results are mainly due to the ditherers of the scales of the regions at which the signals in these two approaches are probed. The typical spot size of the laser beam in Raman investigations is of the order of magnitude of several microns. This allows us to perform precise experimental measurements of the thermal conductivity at the scale of a couple of dozen nanowires. In comparison, the spot in PA measurements is in the range of a few millimetres. Thus, in this case, the signal from a laser area in a Raman-based procedure is acquired, this region contains the nanowires with different morphology and regions with violation of structural perfection. This leads to several tendencies resulting in the smaller measured values of the thermal conductivity in PA measurements. Nevertheless, it should be noted that the magnitudes of the values defining transport properties are scale-dependent and it is essential to consider this for different applications.

Additionally, the Raman experiment often requires significant heating (~ 100 K) for the reliable Raman shift. This may cause completion in analyzing the samples with the significant dependence of thermal conductivity on temperature. At the same time, the heating on PA measurements normally is lower than 1 K.

We performed numerical simulations using the MC-GK method to deepen our understanding of heat transfer phenomena in these doped silicon samples. These simulations allowed us to calculate the thermal conductivities of the samples presented in Table 3. The results obtained are generally in agreement with the experimental measurements.

For case (n° d), an additional diffusion parameter related to an unknown imperfection was taken into account to obtain a satisfactory result. Indeed, this sample has a high electrical resistance (10–20 ohm.cm), which probably caused a defect in the volume structure during its manufacture.

A decrease in thermal conductivity was observed numerically with silicon doping, in agreement with experimental trends. The introduction of dopants creates additional scattering centres, in addition to phonon-phonon, isotope-related scattering, and phonon-boundary interactions, thus reducing the phonon mean free path and, consequently, the thermal conductivity. Numerical simulations have allowed quantifying these effects as a function of the type and concentration of dopants. Table 6 summarizes the available numerical and experimental results concerning the thermal conductivity and the number of processes, classified by type, from numerical simulations involving 500 phonons over 10,000 iterations in three samples: the first in undoped silicon, the second in Sb-doped silicon, and the third in B-doped silicon (additional parameter simulations for a different number of phonons are provided in the supplementary materials, along with graphs showing the thermal conductivity dependence for the undoped, Sb-doped, and B-doped silicon samples). A good agreement is observed between the thermal conductivity results obtained by MC-GK simulation and the experimental data. A significant increase in the percentage of doping-related scattering is observed in the doped silicon samples, which perfectly coincides with the decrease in thermal conductivity observed in these cases. For the films (№ c and № f) of Table 5, a notable effect of porosity on thermal conductivity is observed, attributable to the scattering of phonons by the pores of the structure, which are visible in Fig. 1.

**Table 6:** Thermal conductivities and percentage of diffusions by type in numerical simulations of 500 phonons after 10,000 iterations f.

SiNW- diameter ~ 115 nm	Si - undoped	(b) *n*-Si (y=$\:{5.10}^{-4})$	(c) *n*-Si (y=$\:{5.10}^{-4}$)
MC-GK k(W/$\:{\text{m}}^{-1}{\text{K}}^{-1}$)	43.1 ± 1.1	4.1 ± 0.2	4.3 ± 0.1
Exp. data (ref.) k (W/$\:{\text{m}}^{-1}{\text{K}}^{-1}$)	40 (ref^53^.)	4 ± 1 (Raman technique)	
Border diffusion (%)	48.8	34.4	34.4
Diffusion by isotopes (%)	0.1	0.01	0.01
Diffusion by dopants (%)	0.0	31.0	31.0
Umklapp + Normal (%)	2.4	0.3	0.3

## Conclusions

This study demonstrates the effectiveness of combining photoacoustic and Raman spectroscopy to comprehensively characterize the thermal properties of silicon nanowire arrays and porous silicon-based materials. Our findings reveal that the initial wafer characteristics significantly influence the morphology of these nanostructures, which in turn impacts their thermal conductivity.

While photoacoustic and Raman spectroscopy provide valuable insights, our results highlight the quantitative discrepancies that can arise when using these methods independently. Photoacoustic spectroscopy offers information about the effective thermal conductivity over larger areas, while Raman spectroscopy enables localized measurements. However, the interpretation of experimental data can be influenced by the morphological features of porous silicon, as revealed by our Monte Carlo simulations.

Therefore, a comprehensive approach incorporating both experimental techniques and theoretical modelling is crucial for accurately assessing the thermal transport properties of nanostructured materials. Combining photoacoustic and Raman spectroscopy with Monte Carlo simulations gives us the possibility to understand the factors influencing thermal conductivity in silicon nanowire arrays and porous silicon-based materials.

## Supplementary Information

Below is the link to the electronic supplementary material.

Supplementary Material 1

## Data Availability

The authors declare that the data supporting the findings of this study are available within the paper.
