# Supplementary material for: Peculiarities in thermal transport of nanostructured silicon arrays with different morphology
Source: Sci Rep. 2025 Aug 26;15:31482. doi: 10.1038/s41598-025-13379-4 (PMC12381053; doi:10.1038/s41598-025-13379-4)
Supplement: Supplementary file 1 — Supplementary Material 1 [file 41598_2025_13379_MOESM1_ESM.docx]

Table S1. Thermal conductivities and percentage of diffusions by type in numerical MC -GK simulations (comparison for 500 and 2000 phonons) after 10,000 iterations f.

| Nbre_phonons | 500 | 2000 |
| --- | --- | --- |
| N_ | TC | TC |
| A | 2,9 +-0,1 | 2,9 +- 0,03 |
| B | 4,1 +-0,1 | 4,0 +- 0,05 |
| c P=40% (P=0%) | 3,3 +- 0,1(4,1+-0,1) | 3,4 +-0,1(4,2 +-0,05) |
| D | 7,9 +-0,2 | 8,1 +-0,2 |
| E | 11,7 +-0,2 | 11,8 +- 0,2 |
| f P=40% | 3,9 +-0,3 | 3,7 +-0,1 |


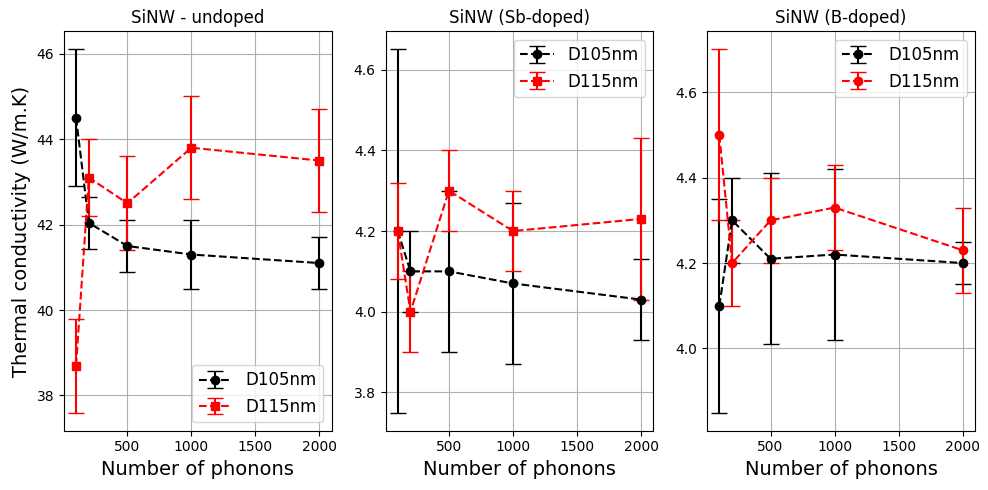


Figure S1. Thermal conductivity of nanowires of diameter equal to 105 nm (black curves) and 115 nm (red curves), with: no doping (left sub-figure), Sb doping (central figure) and B doping (right sub-figure).
